# Influence of dendritic cells on corneal nerve morphological analysis and clinical relevance in chronic dry eye disease after femtosecond laser-assisted laser *in situ* keratomileusis

**DOI:** 10.3389/fmed.2025.1568787

**Published:** 2025-03-31

**Authors:** Yilin Liu, Baikai Ma, Lu Zhao, Hongshuo Li, Wenlong Li, Zhengze Sun, Hongyu Duan, Yitian Zhao, Hong Qi

**Affiliations:** ^1^Department of Ophthalmology, Peking University Third Hospital, Beijing, China; ^2^Beijing Key Laboratory of Restoration of Damaged Ocular Nerve, Peking University Third Hospital, Beijing, China; ^3^Beijing Tongren Eye Center, Beijing Tongren Hospital, Capital Medical University, Beijing Ophthalmic and Visual Science Key Laboratory, Beijing, China; ^4^Cixi Institute of BioMedical Engineering, Ningbo Institute of Materials Technology and Engineering, Chinese Academy of Sciences, Ningbo, China

**Keywords:** dendritic cells, corneal nerve, morphology, FS-LASIK, dry eye disease

## Abstract

**Purpose:**

This study aims to investigate the influence of dendritic cells (DCs) on corneal nerve morphology and the clinical significance in chronic Femtosecond Laser-Assisted Laser *in Situ* Keratomileusis (FS-LASIK) related dry eye disease (DED).

**Methods:**

The cross-sectional study was conducted involving healthy control, DED without FS-LASIK group, and DED after FS-LASIK group. Clinical parameters such as ocular surface disease index (OSDI), fluorescein tear breakup time (FBUT), corneal fluorescein staining (CFS) scores, Schirmer I test (SIt), Cochet-Bonnet esthesiometer (C-BE) were recorded. DCs of *in vivo* confocal microscopy images were included or excluded during corneal nerve segmentation. Key morphological parameters, including corneal nerve fiber density (CNFD), corneal nerve branch density (CNBD), tortuosity, and box-count fractal dimension (Boxdim), were measured. The impact of DCs on nerve metrics and clinical parameters and the correlations between each other were assessed.

**Results:**

The significant reduce in key morphological parameters was observed after eliminating DCs. Significant differences of morphological parameters were observed in DED after FS-LASIK group compared with other two groups. With the increased presence of DCs density in DED especially in DED after FS-LASIK group, the presence of DCs introduced false positives in the correlation analysis of DCs density with corneal morphology in DED after FS-LASIK and in the correlation analysis of corneal morphology with clinical characteristics in DED without FS-LASIK.

**Conclusion:**

The presence of DCs introduces significant biases in the assessments of corneal nerve morphology, primarily false-positive results in DED especially chronic FS-LASIK related DED. Their exclusion improves the precision of nerve measurements, which may enhance the clinical evaluation of corneal nerve morphology. These findings highlight the importance of precise segmentation techniques to minimize DCs related interference in clinical practice.

## 1 Introduction

In recent decades, femtosecond laser-assisted *in situ* keratomileusis (FS-LASIK) has become the most commonly performed corneal refractive surgery (1). A majority of patients experience some form of discomfort associated with dry eye disease (DED) during the postoperative period (2). While DED typically manifests transiently in the early postoperative phase, approximately 8 to 20% of patients exhibit chronic DED lasting more than 6 months (1, 3, 4). Refractive surgery-induced damage to corneal nerves plays a critical role in the development of DED (5, 6). Structural changes in corneal nerves, such as thinning, increased tortuosity, or loss of fibers, serve as early indicators of ocular surface issues (7, 8). Therefore, analysis of these changes is essential for understanding DED. Normally, the cornea hosts a limited number of resting dendritic cells (DCs) (9). In pathological states of DED, there is a significant increase in activated DCs, contributing to ocular inflammation (10, 11). Activated DCs engage in structural interactions with corneal nerves and exert functional impacts on nerve regeneration (12, 13). However, the precise nature of this interaction, particularly in the context of DED, remains to be elucidated.

Confocal microscopy has become an essential tool for evaluating corneal nerve structure and DCs *in vivo* (14). Artificial intelligence has significantly advanced our understanding of corneal neurology under normal and pathological conditions. However, distinguishing between corneal nerves and DCs remains challenging due to their similar refractive properties, which complicates the accurate assessment of nerve morphology (8, 15). The impact of DCs on nerve imaging, along with their clinical significance—especially in diagnosing and managing DED—requires further investigation.

In this study, we aim to examine how DCs influence corneal nerve structure and clarify clinical biases in chronic FS-LASIK related dry eye. Additionally, we introduce an artificial intelligence-based system for autonomous segmentation of corneal nerves and DCs, improving morphological analysis accuracy. This innovation enhances DED diagnosis precision and deepens our understanding of the disease’s pathophysiology.

## 2 Methods

### 2.1 Patients

This cross-sectional study was conducted from October 2021 to September 2022 at the Department of Ophthalmology, Peking University Third Hospital. Participants who experienced persistent DED after undergoing FS-LASIK for 1 year were included in the DED after FS-LASIK group. The inclusion criteria were as follows: continuous DED symptoms for a minimum of 6 months after FS-LASIK and no history of DED symptoms or signs prior to the procedure. Participants with DED without FS-LASIK were enrolled in the DED without FS-LASIK group and participants without dry eye and without FS-LASIK were enrolled in the control group. Participants were diagnosed with DED according to the Dry Eye Workshop (DEWS) II criteria (16). Exclusion criteria included individuals with a history of any other ocular surface disease, those who failed to discontinue contact lens use for at least 2 weeks prior to the study, those using any medications other than artificial tears within 2 weeks before enrollment, and individuals with systemic disorders affecting the ocular surface, such as Sjögren’s syndrome. This study was approved by the Ethics Committee of Peking University Third Hospital (M2021391) and adhered to the principles of the Declaration of Helsinki. Written informed consent was obtained from all participants.

### 2.2 Surgical technique

All FS-LASIK procedures were performed by an experienced surgeon under topical anesthesia. Corneal flaps were created using a WaveLight FS200 femtosecond laser (Alcon Laboratories, Inc.) with a pulse energy of 0.80 μJ, a thickness of 110 μm, and a diameter of 8.5 to 9 mm. The hinge position was superior. Laser ablation was conducted using a WaveLight EX500 excimer laser (Alcon Laboratories, Inc.) with an optical zone diameter of 6 to 6.5 mm. The target postoperative refraction was emmetropia in all eyes. All surgical procedures were completed without complications.

### 2.3 Ocular surface evaluation

DED symptoms were evaluated using the Ocular Surface Disease Index (OSDI) questionnaire (17). All clinical examinations were carried out by a single examiner for each patient. To evaluate the fluorescein tear breakup time (FBUT), a fluorescein strip, pre-moistened with an antiseptic saline solution, was placed in the inferior conjunctival fornix. Participants were subsequently observed under a slit-lamp biomicroscope equipped with a cobalt blue filter. The average of three measurements was documented. Corneal fluorescein staining (CFS) scores were evaluated according to the National Eye Institute Workshop guidelines (total score: 0 to 15) (18). The Schirmer I test (SIt) was conducted using Schirmer paper strips (5 × 35 mm) without anesthesia. Corneal sensitivity was assessed with a Cochet-Bonnet esthesiometer (C-BE; Luneau Technology Operations SAS, France) equipped with a 6-cm adjustable nylon monofilament, which was progressively shortened in 5-mm increments until the first response was observed. Greater corneal sensitivity is indicated by longer filament lengths (19). The right eye of each patient was selected for analysis. The examinations for each patient were conducted in the sequence described above, and the following invasive examination was then carried out.

### 2.4 Image acquisition

The imaging of corneal nerve was obtained through a laser scanning *in vivo* confocal microscope (IVCM) (Heidelberg Engineering GmbH, Heidelberg, Germany) using the Heidelberg Retina Tomograph 3 with the Rostock Cornea Module. After administering topical anesthesia, 0.2% carboxypolymethylene gel was applied to both the ocular surface and the external tip of the device’s cap to improve optical coupling. Image acquisition was performed at a depth of 40 to 60 μm beneath the anterior surface of the central cornea. Each image featured a resolution of 384 × 384 pixels and spanned an area of 400 × 400 μm^2^. For further analysis, images were chosen from the right eye, avoiding the whorl region.

### 2.5 Automatic segmentation of corneal nerves and DCs

We present a novel end-to-end encoder-decoder architecture aimed at achieving precise segmentation of corneal nerves and DCs. The proposed model utilizes a pretrained ResNet50 as its backbone and incorporates two specialized decoder branches for targeted feature extraction specific to each structure. For corneal nerve segmentation, the decoder employs layers constructed using the Multi-Scale Fusion Module (MSFM), which improves feature representation across scales. However, the ambiguous boundaries of DCs in IVCM images pose a significant challenge for accurate segmentation. To address this issue, we propose the Uncertainty Boundary Guided Module (UBGM), an innovative mechanism based on uncertainty-based attention. The UBGM processes features extracted by the encoder to generate uncertainty maps at each resolution recovery stage. These maps are normalized and then binarized to create uncertainty masks. These masks are used to weight the encoder features, thereby refining feature extraction through the integration of uncertainty-guided attention and the Convolutional Block Attention Module (CBAM). This integrated approach significantly improves feature discrimination, effectively addressing the inherent challenges posed by the ambiguous boundaries of DCs during segmentation.

### 2.6 Statistical analysis

Prism 8.0 (GraphPad, Boston, MA, United States) was used for statistical analysis. The normality assumption was checked with the Shapiro–Wilk test. Quantitative data were described using mean ± standard deviation as appropriate. The Chi-square test was used to examine sex differences between groups. For the comparation of three groups, when data were normally distributed and homogeneity of variance, the one-way analysis of variance was conducted with the Bonferroni’s multiple comparisons test for post-hoc comparisons. When data were normally distributed and the homogeneity of variances assumption is not met, Welch’s test was conducted with the Tamhane’s T2 multiple comparisons test for post-hoc comparisons. When data were non-normally distributed, Kruskal-Wallis test was conducted with the Dunn’s multiple comparisons test for post-hoc comparisons. The Wilcoxon matched-pairs signed rank test was performed in the analysis of system data sets. The Spearman’s correlation coefficient was applied to explore the between variable correlations. A *p* value less than 0.05 was considered statistically significant.

## 3 Results

### 3.1 Demographic and clinical characteristics among subjects

This study enrolled 61 participants (61 eyes), including 17 patients with chronic DED after undergoing FS-LASIK, 20 patients with DED without FS-LASIK, and 24 normal controls. No statistically significant differences were found in sex or age among the three groups (both *p* > 0.05) (Table 1).

**Table 1:** Demographic characteristics of the study groups.

Characteristics	DED after FS-LASIK	DED without FS-LASIK	Control	*p*-value
No. of participants/eyes	17/17	20/20	24/24	-
No. male/female	2/15	5/15	10/14	0.103
Age (mean ± SD, years)	30.53 ± 5.84	26.35 ± 5.86	26.88 ± 7.18	0.078

DED, dry eye disease; FS-LASIK, femtosecond laser-assisted laser in situ keratomileusis; SD, standard deviation.

Compared to the control group, both DED groups had higher OSDI, CFS scores and lower FBUT (all *p* < 0.05). No statistically significant differences were observed between the DED after FS-LASIK group and the DED without FS-LASIK group in terms of OSDI, FBUT, or CFS scores. The DED after FS-LASIK group showed significantly lower values for the SIt than the control group (*p* < 0.05). Both the DED after FS-LASIK group and the control group showed significantly higher values for the C-BE test than the DED without FS-LASIK group (both *p* < 0.001) and no statistically significant differences were observed between the DED after FS-LASIK group and the control group (Table 2).

**Table 2:** Clinical characteristics of the study groups.

Characteristics	DED after FS-LASIK	DED without FS-LASIK	Control	*p*-value (1 vs 2)	*p*-value (1 vs 3)	*p*-value (2 vs 3)
OSDI score (mean ± SD)	19.01 ± 4.61	32.09 ± 13.01	5.19 ± 4.22	0.093	<0.001	<0.001
FBUT (mean ± SD, s)	3.06 ± 1.35	4.20 ± 1.51	11.38 ± 4.00	0.385	<0.001	<0.001
CFS score (mean ± SD)	1.88 ± 2.52	2.30 ± 1.69	0.00 ± 0.00	0.461	0.003	<0.001
SIt (mean ± SD, mm)	9.00 ± 4.72	15.05 ± 12.28	19.13 ± 9.87	0.598	0.019	0.430
C-BE (mean ± SD, cm)	6.00 ± 0.00	5.91 ± 0.10	5.96 ± 0.14	<0.001	>0.999	<0.001

OSDI, Ocular Surface Disease Index; FBUT, Fluorescein breakup time; CFS, Corneal fluorescein staining; SIt, Schirmer I test; C-BE, Cochet-Bonnet esthesiometer.

### 3.2 Change in corneal nerve morphological features upon exclusion of DCs

**Figure 1:**
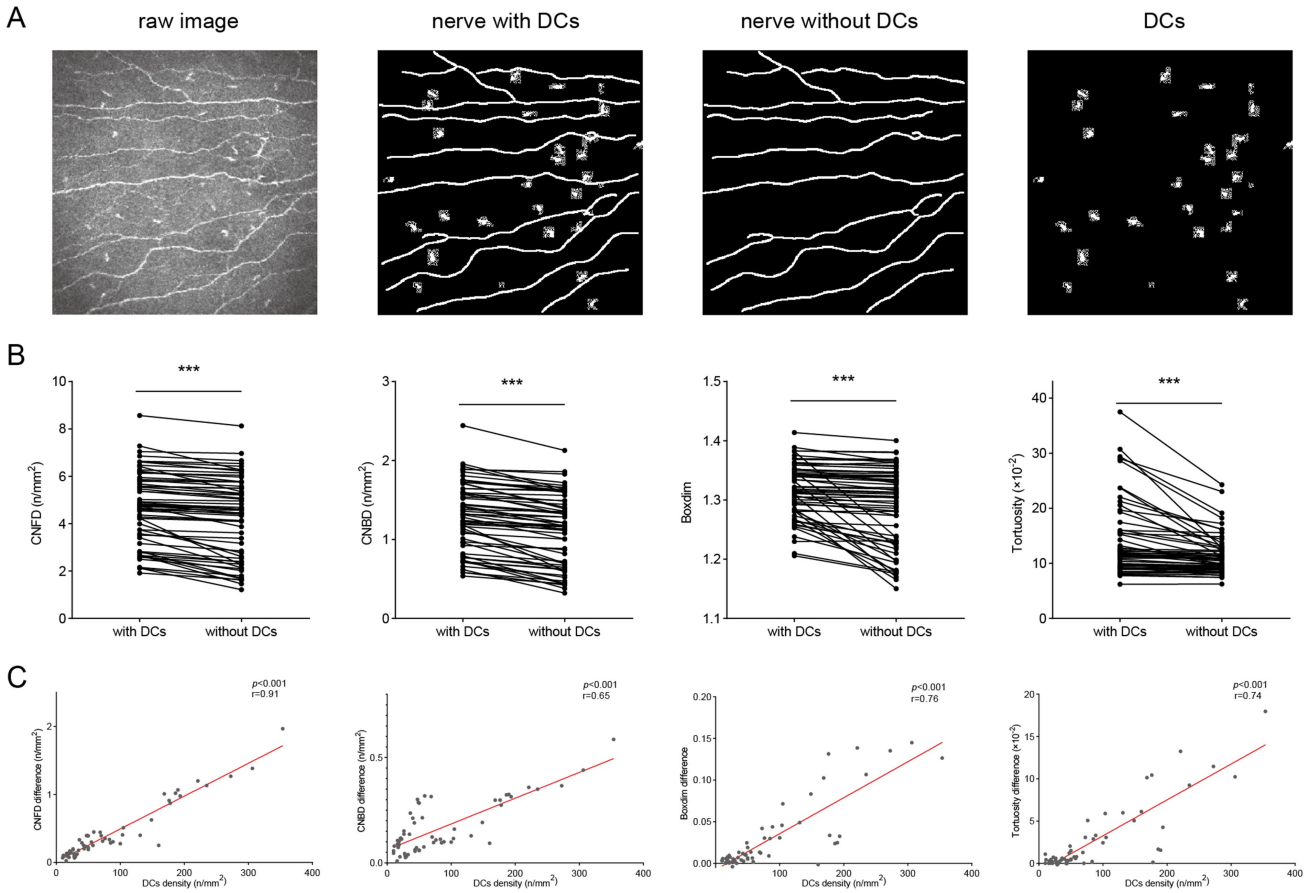
Impact of DCs on corneal nerve morphology and quantitative metrics. **(A)** Representative raw corneal nerve images and their segmentations (nerve segmentation with DCs, nerve segmentation without DCs, and segmented DCs alone); **(B)** The differences in CNFD, CNBD, boxdim, and tortuosity when DCs were included and excluded; **(C)** Correlations of DCs density with differences in CNFD, CNBD, boxdim, and tortuosity. DCs, dendritic cells; CNFD, corneal nerve fiber density; CNBD, corneal nerve branch density; boxdim, box-count fractal dimension. ^***^p < 0.001.

Images from three groups were analyzed together to evaluate the influence of DCs on corneal nerve morphology. Representative raw images and segmentation results were shown in Figure 1A. After excluding DCs, the morphological features of corneal nerves detected by confocal microscopy were significantly reduced (Figure 1B). The corneal nerve fiber density (CNFD) decreased substantially after DCs were excluded (4.69 ± 1.54 /mm2 vs. 4.29 ± 1.69 /mm2, *p* < 0.001). The corneal nerve branch density (CNBD) exhibited a marked reduction following DCs exclusion (1.29 ± 0.43 /mm2 vs. 1.13 ± 0.45 /mm2, *p* < 0.001). The box-count fractal dimension (Boxdim) showed a slight decrease when DCs were excluded (1.32 ± 0.05 vs. 1.29 ± 0.07, *p* < 0.001). The tortuosity was significantly reduced following DCs exclusion (13.78 ± 6.68 × 10–2 vs. 11.34 ± 3.55 × 10–2, *p* < 0.001) (Figure 1B). Furthermore, the differences in CNFD, CNBD, boxdim and tortuosity (characteristics of segmented images containing DCs minus those excluding DCs) were positively correlated with the density of DCs (all *p* < 0.001) (Figure 1C).

### 3.3 Corneal nerve morphology analysis with the inclusion and exclusion of DCs

**Figure 2:**
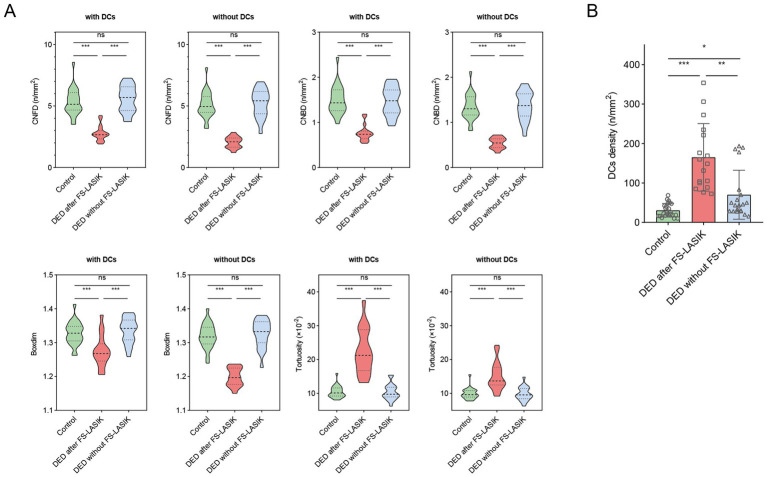
Comparisons of corneal nerve parameters and DCs among study groups. **(A)** Violin plots depicting CNFD, CNBD, boxdim, and tortuosity among the control group, DED after FS-LASIK group, and DED without FS-LASIK group. For all metrics, results were shown both with DCs inclusion and exclusion; **(B)** The differences in DCs density among the control group, DED after FS-LASIK group, and DED without FS-LASIK group. ^*^p < 0.05; ^**^p < 0.01; ^***^p < 0.001; ns, no significance.

This study examined corneal nerve morphological characteristics using segmented images, both including and excluding DCs (Figure 2A). For CNFD, CNBD and boxdim, there was no change in significant difference with or without DCs included in the analysis, that is, values in DED after FS-LASIK group were significantly lower than those in control group and DED without FS-LASIK group (all *p* < 0.001), and there was no significant difference between the latter two groups (all *p* > 0.05). For tortuosity, values in DED after FS-LASIK group were significantly higher than that in control group and DED without FS-LASIK group (all *p* < 0.001), and this trend of disparity remained unchanged whether DCs were included or excluded.

We further analyzed DCs density among the three groups (Figure 2B). The DCs density of control group was significantly lower than that of two DED groups (both *p* < 0.05). The DCs density of DED after FS-LASIK group was significantly higher than that of the DED without FS-LASIK group (*p* < 0.01).

### 3.4 Correlations between DCs density and clinical characters

**Figure 3:**
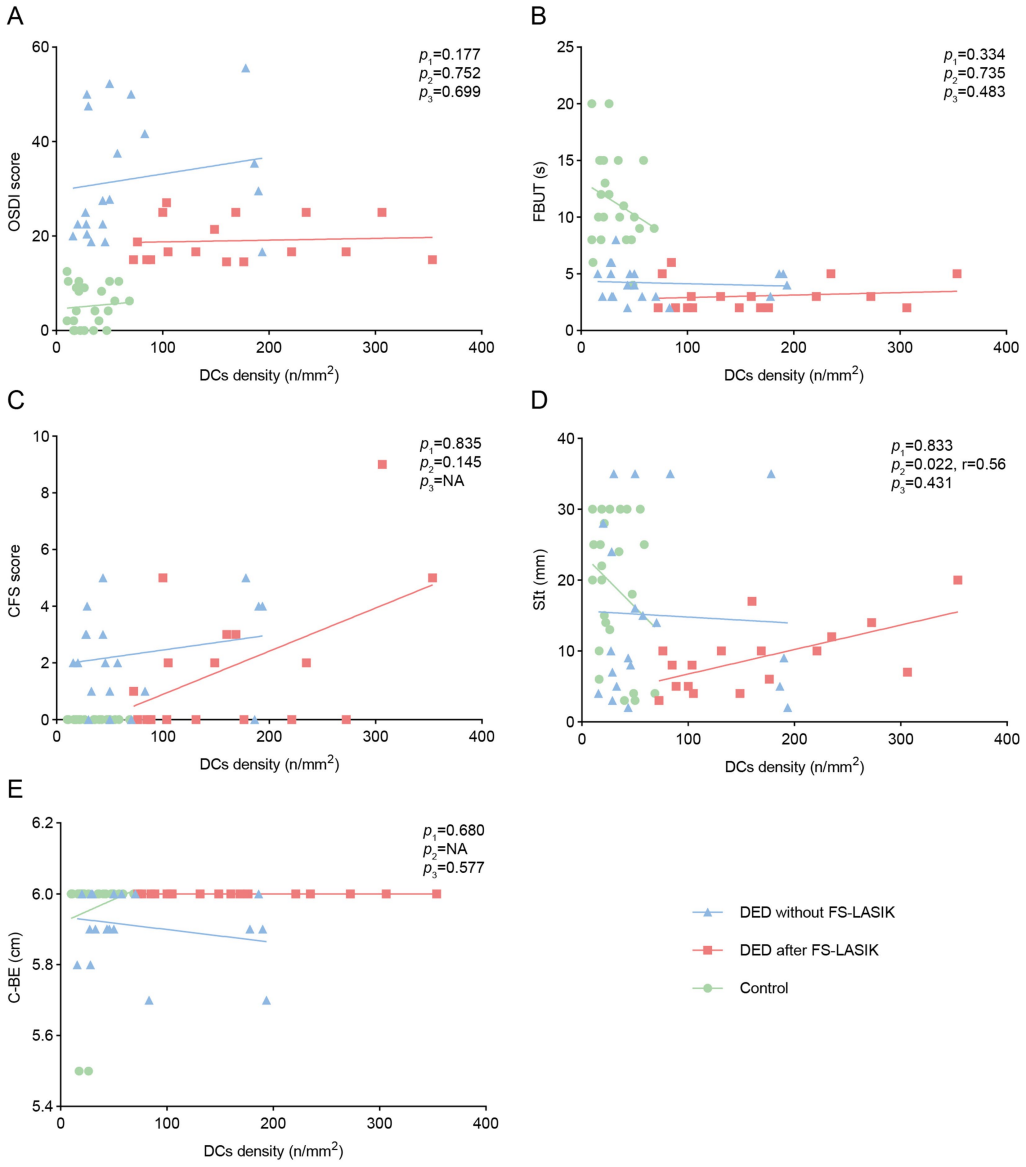
Correlations of DCs density with ocular surface symptoms and signs among study groups. Correlations of DCs density with **(A)** OSDI, **(B)** FBUT, **(C)** CFS, **(D)** SIt, **(E)** C-BE among the control group, DED after FS-LASIK group, and DED without FS-LASIK group. p1, the *p* value of DED without FS-LASIK group; p2, the *p* value of DED after FS-LASIK group; p3, the *p* value of control group; NA, not available.

We explored the correlations between DCs density and clinical characters (OSDI, FBUT, SIt, CFS scores and C-BE) in the control group, DED after FS-LASIK group, and DED without FS-LASIK group, respectively (Figure 3). In the control group and DED without FS-LASIK group, DCs density was not significantly correlated with any clinical indicators (all *p* > 0.05). In DED after FS-LASIK group, DCs density was significantly correlated with SIt (*r* = 0.56, *p* = 0.022) (Figure 3D) but not with other clinical indicators (all *p* > 0.05).

### 3.5 Correlations between DCs density and corneal nerve morphological features

**Figure 4:**
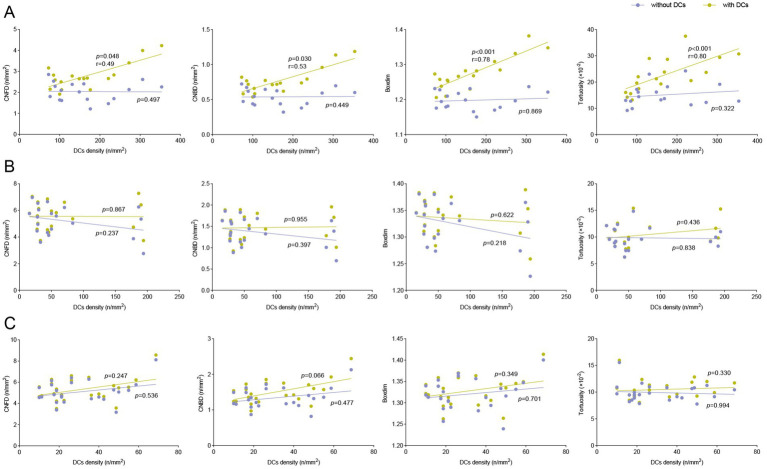
Correlations of DCs density with corneal nerve morphology and quantitative metrics in study groups. Correlations of DCs density with CNFD, CNBD, boxdim, and tortuosity when DCs were included and excluded among **(A)** DED after FS-LASIK group, **(B)** DED without FS-LASIK group and **(C)** control group.

We explored the correlations between DCs density and corneal nerve morphological features (CNFD, CNBD, boxdim, and tortuosity) in the control group, DED after FS-LASIK group, and DED without FS-LASIK group, respectively. In DED after FS-LASIK group, when DCs were included, DCs density was significantly correlated with CNFD (*r* = 0.49, *p* = 0.048), CNBD (*r* = 0.53, *p* = 0.030), boxdim (*r* = 0.78, *p* < 0.001), and tortuosity (*r* = 0.80, *p* < 0.001). When DCs were excluded, DCs density was not significantly correlated with any corneal nerve morphological features (all *p* > 0.05) (Figure 4A). In DED without FS-LASIK group and control group, DCs density was not significantly correlated with any corneal nerve morphological features when DCs were included or excluded (all *p* > 0.05) (Figures 4B,C).

### 3.6 Correlations between corneal nerve morphology and clinical characters

**Figure 5:**
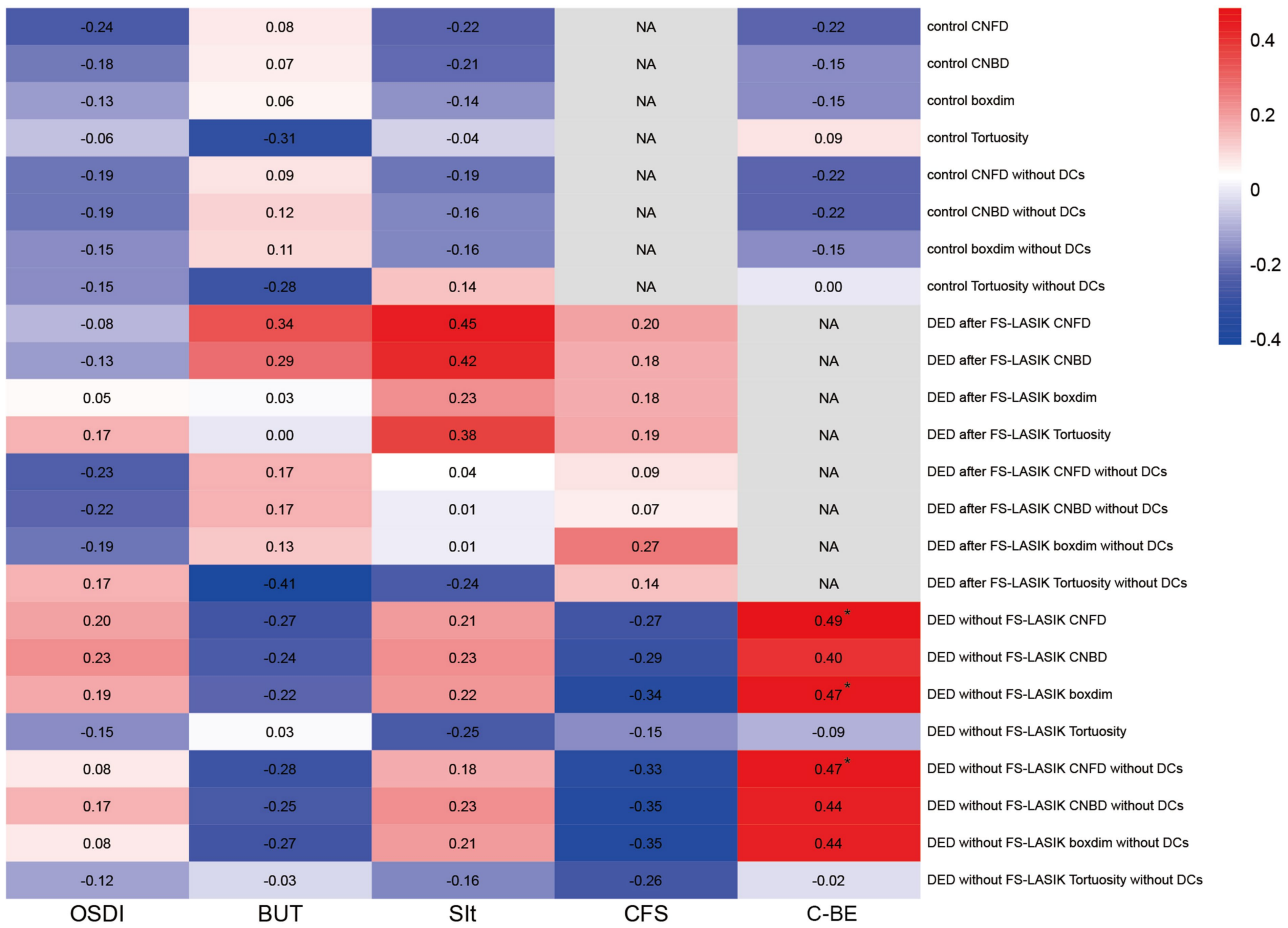
Correlations of ocular surface symptoms and signs with corneal nerve morphology and quantitative metrics in study groups. The heatmap shows the value of the correlation coefficient between ocular surface symptoms and signs (OSDI, FBUT, CFS, SIt, C-BE) with corneal nerve morphology and quantitative metrics (CNFD, CNBD, boxdim, and tortuosity) when DCs were included and excluded among the control group, DED after FS-LASIK group, and DED without FS-LASIK group. ^*^p < 0.05. NA, not available.

To explore the clinical relevance of DCs-induced biases in corneal nerve morphology analysis, we assessed correlations between clinical parameters (OSDI, FBUT, SIt, CFS scores and C-BE) and corneal nerve morphological features (CNFD, CNBD, boxdim, and tortuosity), both with and without DC inclusion (Figure 5). In the control group and DED after FS-LASIK group, there were no significant correlations between clinical parameters and corneal nerve morphological features with the inclusion and exclusion of DCs (all *p* > 0.05). In DED without FS-LASIK group, when DCs were included, C-BE was significantly correlated with CNFD (*r* = 0.49, *p* = 0.030) and boxdim (*r* = 0.47, *p* = 0.037). When DCs were excluded, C-BE was only significantly correlated with CNFD (*r* = 0.47, *p* = 0.039). There were no significant correlations between other clinical parameters and corneal nerve morphological features in DED without FS-LASIK group (all *p* > 0.05).

## 4 Discussion

This study examines the impact of interplay between DCs and corneal nerve morphology, it is the first study to investigate the influence of DCs on corneal nerve morphological analysis and clinical characteristics analysis in patients with DED, particularly chronic FS-LASIK-related DED.

In our study, the subjective symptoms and objective clinical indicators in the two DED groups were similar, indicating that the severity of dry eye in these two groups was comparable. The only difference in SIt between DED after FS-LASIK group and the control group might be due to the variations not required by the inclusion criteria (16). For corneal sensitivity measured by C-BE, the DED without FS-LASIK group was less sensitive than the other two groups. There are conflicting results regarding the changes in corneal sensitive in dry eye patients. Some studies have demonstrated a decline in corneal sensation (20–22), while other studies have shown increased corneal sensitivity (23, 24). Besides, other clinical studies have shown that corneal sensitivity recovers to the preoperative level within 3 to 16 months after LASIK (5, 25). The variability of results in the corneal sensitivity may be attributed to the level of corneal inflammation in the enrolled patients.

Dendritic cells are bone marrow derived professional antigen presenting cells that populate the interstitial tissue of most solid organs including the ocular surface. These cells play an important role in both the innate and adaptive arms of the immune system (26, 27). Our research and previous studies have found an increase in the corneal DCs density in patients with DED (28–30). Studies have already described a possible role for corneal DCs in the etiopathogenesis of dry eye, keratoconjunctivitis sicca, and corneal allograft rejection (31, 32). The density of DCs in the DED after FS-LASIK group was the highest among three groups. Given that DCs are immune cells and are related to inflammation (33), this phenomenon may be associated with some chronic inflammation after surgery, which requires further investigation. Studies have observed that DCs are often positioned in close proximity to corneal sensory nerves, the DCs were situated in the sub-basal space adjacent to the nerve plexus, with their dendrites crossing several nerve endings (12). With the increased density of DCs in DED especially in FS-LASIK related DED and the similarity of the morphology of DCs and corneal nerves, this might influence the accurate analysis of the morphology of corneal nerves. It is important to eliminate the confusion caused by DC in corneal morphology analysis.

The ACCMetrics software has played a pivotal role in advancing the quantitative analysis of corneal nerve morphology and remains widely utilized (34). However, co-segmentation of corneal nerves and DCs when applying ACCMetrics suggested that DCs may interfere with the accurate analysis of nerve morphology. Given the frequent presence of DCs in inflammatory conditions of the ocular surface and even in healthy individuals, it is imperative to investigate the specific impact of DCs on nerve morphological parameters. Recent progress in automated segmentation of IVCM images, such as the work by Zemborain et al. (35), have initiated precise quantification of corneal nerves by classifying structures like nerves, neuromas, and immune cells. This model directly segments enhanced images, demonstrating robustness in handling extremely poor quality images by eliminating artifacts and noise. In contrast, our approach leverages prior knowledge of the topological structure of nerve fibers to enhance segmentation continuity and utilizes the uncertainty of cell boundary segmentation as a feedback mechanism to achieve more accurate boundary delineation. These refinements provide a significant improvement in the accuracy of corneal nerve analysis and highlight the necessity of addressing DCs related interference for reliable morphological assessments.

Corneal nerve parameters, including CNFD, CNBD and tortuosity, have been established as sensitive indicators of neuropathy in ocular surface disorders, including DED (8, 36–38) and other systemic diseases (39, 40). Boxdim reflects the complexity of nerves. Our data demonstrate a reduction in CNFD, CNBD, boxdim and tortuosity following the exclusion of DCs, and the reduction extent of these metrics correlated significantly with the DCs density, indicating the potential overestimation of these parameters under inflammatory conditions. These results suggested that, while DCs play a crucial role in neuroimmune interactions as highlighted in previous studies (41, 42), they also inadvertently introduce substantial variability into corneal nerve measurements. This finding highlights the importance of evaluating the impact of DCs on corneal nerve morphology, particularly in inflammatory states.

For the specific intergroup analysis of each corneal morphology index, we observed that, irrespective of DCs removal, no significant differences were noted in any corneal indices between the control group and DED without FS-LASIK group. Compared with these two groups, in DED after FS-LASIK group, CNBD, CNFD and boxdim were markedly reduced, while tortuosity was significantly elevated, which is consistent with previous literature (38, 43). This indicates that the differences in these corneal morphology metrics among these groups are significant enough to ignore the impact brought by DCs, which also fully demonstrates the influence of FS-LASIK on corneal nerves. Due to the need to make an incision or corneal flap on the cornea, refractive surgery will inevitably damage the corneal nerves. The lower the corneal nerve density, the more the degree of corneal nerve damage (44). Researchers suggested that the corneal nerve damage caused by FS-LASIK could not be repaired to the same level as that before FS-LASIK even 10 years postoperatively (45). When conducting a correlation analysis between the various indicators of the cornea morphology with the DCs density among three groups, we observed significant changes in DED after FS-LASIK group. After excluding DCs, the significant correlation between DCs density and all indicators became statistically insignificant, indicating that the inclusion of DCs brought about false positive results in this analysis. It had little impact on other groups, possibly because there were fewer DCs in other groups.

False positives were also found in the correlation analysis of clinical features and corneal morphology indicators in DED without FS-LASIK group. When DCs were excluded, the number of corneal morphological parameters related to corneal sensitivity that is measured by C-BE decreased from 2 to 1, and the correlation coefficient also slightly declined. Studies have reported that corneal sensitivity was correlated with the density and the number of corneal nerves (22, 46). There are also studies reported the correlation of other clinical features such as Schirmer test and CFS scores with corneal morphology metrics (47–49). These differences may be attributed to the variations in the analyzed populations and analytical methods, as well as the inability to eliminate the influence of DC. We also found that DCs density was only significantly correlated with SIt in DED after FS-LASIK group. Studies have reported the correlations between DCs density with Schirmer test and tear breakup time in dry eye patients (10, 50). These discrepancies may be due to the different participants enrolled and DED after FS-LASIK group showed most DCs density and relatively low SIt.

Our research has several limitations. Firstly, although IVCM technology offers high-resolution imaging, it is constrained by a limited field of view and may be affected by operator variability. Secondly, this study primarily examined the structural relationship between DCS and corneal nerves without exploring their functional interactions in clinical contexts. Future studies should aim to investigate these functional aspects to enhance our understanding of neuroimmune interactions in ocular surface diseases. Lastly, expanding the study to include a more diverse cohort could strengthen the generalizability of the findings. Extending studies to encompass other types of dry eye conditions could elucidate whether the observed phenomena are specific to FS-LASIK related dry eye or represent a broader pattern of neuroimmune interactions across various ocular surface disorders.

This study offers valuable insights into the intricate interaction between DCs and corneal nerve morphology, first underscoring the significant impact of DCs on the interpretation of corneal nerve parameters in FS-LASIK-associated DED. By eliminating DCs interference, the significant reduce in key morphological parameters was observed. With the increased presence of DCs density in DED especially in FS-LASIK-associated DED, the presence of DCs introduces potential false positives in the correlation analysis of corneal morphology and clinical characteristics. These findings highlight the necessity of employing precise segmentation techniques to minimize immune cell interference and enhance our understanding of neural-immune interactions in dry eye, and provide a foundation for developing accurate diagnostic and therapeutic strategies. Future studies should further investigate the mechanisms between DCs and neurons and their potential implications in ocular diseases.

## Data Availability

The datasets presented in this article are not readily available because the datasets generated and/or analyzed during the current study are not publicly available due to privacy concerns related to patient data. Access to these data can be provided upon reasonable request to the corresponding author, subject to ethical approval and data protection regulations. Requests to access the datasets should be directed to doctorqihong@163.com.
